# OsVPE2, a Member of Vacuolar Processing Enzyme Family, Decreases Chilling Tolerance of Rice

**DOI:** 10.1186/s12284-023-00682-9

**Published:** 2024-01-09

**Authors:** Huabing Deng, Sai Cao, Guilian Zhang, Yunhua Xiao, Xiong Liu, Feng Wang, Wenbang Tang, Xuedan Lu

**Affiliations:** 1 College of Agronomy, Hunan Agricultural University, Changsha, 410128 China; 2 State Key Laboratory of Hybrid Rice, Hunan Hybrid Rice Research Center, Hunan Academy of Agricultural Sciences, Changsha, 410125 China; 3 Yuelushan Laboratory, Changsha, 410128 China

**Keywords:** Rice, Chilling, Vacuolar Processing Enzyme, Reactive Oxygen Species, Natural Variation

## Abstract

Chilling is a major abiotic stress affecting rice growth, development and geographical distribution. Plant vacuolar processing enzymes (VPEs) contribute to the seed storage protein processing and mediate the programmed cell death by abiotic and biotic stresses. However, little is known about the roles of plant VPEs in cold stress responses and tolerance regulation. Here, we found that *OsVPE2* was a chilling-responsive gene. The early-*indica* rice variety Xiangzaoxian31 overexpressing *OsVPE2* was more sensitive to chilling stress, whereas the *OsVPE2-*knockout mutants generated by the CRISPR-Cas9 technology exhibited significantly enhanced chilling tolerance at the seedling stage without causing yield loss. Deficiency of *OsVPE2* reduces relative electrolyte leakage, accumulation of toxic compounds such as reactive oxygen species and malondialdehyde, and promotes antioxidant enzyme activities under chilling stress conditions. It was indicated that *OsVPE2* mediated the disintegration of vacuoles under chilling stress, accompanied by the entry of swollen mitochondria into vacuoles. *OsVPE2* suppressed the expression of genes that have a positive regulatory role in antioxidant process. Moreover, haplotype analysis suggested that the natural variation in the *OsVPE2* non-coding region may endow *OsVPE2 *with different expression levels, thereby probably conferring differences in cold tolerance between *japonica* and *indica* sub-population. Our results thus reveal a new biological function of the VPE family in regulating cold resistance, and suggest that the gene editing or natural variations of *OsVPE2* can be used to create cold tolerant rice varieties with stable yield.

**Supplementary Information:**

The online version contains supplementary material available at 10.1186/s12284-023-00682-9.

## Introduction

Rice (*Oryza sativa* L.) is one of the most important staple food crops for over half of the world’s population. Recently, the rice production has been threatened by abnormal environmental temperatures induced by global climate change (Zhang et al. 2021). Rice is originated in tropics and subtropics and subsequently expands to a wide range of geographical regions (Huang et al. 2012). With its thermophilic characteristics, rice is sensitive to chilling stress, which adversely affects its growth, development and yield. For example, early-*indica* rice in Double-Crop rice production in South China are prone to chilling stress at the seedling stage, which leads to wilting, yellowing, and decreased survival rates (Cheng et al. 2013). Identifying chilling responsive genes at seedling stage will help cultivate chilling-tolerant rice varieties, ensuring yield safety.

Chilling stress induces a series of physical damages and physiological reactions in rice. Chilling damage to membrane lipids leads to lipid peroxidation and electrolyte leakage (EL) (Morsy et al. 2007; Song et al. 2011; Lukatkin et al. 2012). Moreover, chilling stress alters the homeostasis of reactive oxygen species (ROS), which are signaling molecules induced by stresses and trigger further defense responses. However, excessive ROS accumulation due to prolonged chilling can cause oxidative damage (Gill and Tuteja 2010). Excessive ROS further induce the generation of reactive carbonyl species (RCS) such as malondialdehyde (MDA), which are toxic to cells through lipid peroxides (Mano 2012). To scavenge ROS, enzymatic antioxidants such as superoxide dismutase (SOD) and peroxidase (POD), non-enzymatic antioxidants such as ascorbic acid and glutathione, as well as flavonoids and anthocyanins are produced (Mittler et al. 2011; Shi et al. 2020; Islam et al. 2023). The catalase coding gene *OsCATB* (*CATALASE ISOZYME B*) is mainly involved in the oxidative stress response of rice and positively regulates the oxidative stress tolerance of rice (Ye et al. 2011; Ni et al. 2022). The superoxide dismutase encoded by the *Fe*^*+*^*-SOD* (*IRON SUPEROXIDE DISMUTASE*) gene is an important antioxidant enzyme that balances oxygen free radicals and plays an important role in scavenging ROS (Kaminaka et al. 1999). The ascorbic acid peroxidase encoded by *OsAPX1* (*ASCORBATE PEROXIDASES*), is a crucial enzyme to detoxify ROS in rice by catalyzing the conversion of H_2_O_2_ into H_2_O (Rosa et al. 2010). *SNAC1* (*STRESS-RESPONSIVE NAC1*) promotes oxidative stress tolerance through abscisic acid-independent ROS scavenging in rice (You et al. 2014).

Chilling tolerance in rice is a quantitative trait that is controlled by many genes (Li et al. 2022). Over the past few decades, more than 250 quantitative trait loci (QTL) related to chilling tolerance in rice have been studied, although lots of them have been only roughly mapped (Mao et al. 2015; Endo et al. 2016; Li et al. 2022). The chilling tolerance divergence 1 (COLD1) interacts with the G-protein α subunit 1 (RGA1) to trigger calcium (Ca^2+^) signaling for sensing low temperature (Ma et al. 2015). Under chilling stress, cold-activated OsMAPK3 phosphorylates the transcription factor OsbHLH002 and prevents it from degradtion. Phosphorylated OsbHLH002 directly activates *OsTPP1* transcription, leading to accumulation of the osmoprotectant trehalose and increased chilling tolerance (Zhang et al. 2017). In addition, the cyclic nucleotide-gated channel OsCNGC9 triggers an increase in cytosolic Ca^2+^ levels, which in turn, activates chilling responsive genes and thus enhances chilling tolerance in rice (Wang et al. 2021). The natural variations of *COLD1, COLD11*, *COG2*, *bZIP73*, *HAN1*, *LTG1*, *CTB4a*, *CTB2*, *OsMAPK3* and *OsLEA9*, endow rice with chilling resilience (Lu et al. 2014; Ma et al. 2015; Zhang et al. 2017; Liu et al. 2018, 2019; Mao et al. 2019; Li et al. 2021, 2023; Lou et al. 2022; Feng et al. 2023). Moreover, several subfamilies of transcription factors including the CBFs/DREBs (C-repeat-binding factors/dehydration-responsive element-binding proteins), MADS (MCM1, Agamous, Deficiens, and Serum response factor, WRKY, MYB (myeloblastosis), bHLH, NAC, TCP (Teosinte branched1/Cincinnata/proliferating cell factor), etc., respond to chilling stress and mediate the regulatory network of chilling tolerance (Dubouzet et al. 2003; Huang et al. 2016; Lv et al. 2017; Zhang et al. 2017, 2022, 2023; Chen et al. 2018; Li et al. 2022).

Plant vacuolar processing enzymes (VPEs), also known as legumain or asparaginyl endopeptidase (AEP), were named because of their roles in the proteolytic processing of various vacuolar proteins (Hara-Nishimura and Nishimura 1987). VPEs have been classified into three types based on the expression and functional characteristics: the vegative organ type (for example, αVPE and γVPE of *Arabidopsis thaliana*), the embryo type (for example, βVPE of *Arabidopsis*) and the seed coat type (for example, δVPE of *Arabidopsi*s) (Kinoshita et al. 1995a, b; Nakaune et al. 2005). However, this grouping does not exclude their expression and activity in other tissues or developmental stages, which has been revealed by further studies in many species such as *Arabidopsis*, tobacco, barley and so on (Shimada et al. 2003; Zakharov and Muntz 2004; Julian et al. 2013). VPEs with caspase-1 activity contribute to the release of hydrolytic enzymes to the cytosol from the collapsed vacuole during programmed cell death (PCD) induced at various developmental stages or by stresses (Hatsugai et al. 2004, 2015). Thus, VPEs play important roles in the regulation of development (seed coat formation, pollen self-incompatibilities, etc.), biotic stress response (pathogens, elicitors, toxins, etc.) and abiotic stress response (heat, hydrogen peroxide, osmotic change, aluminium, etc.) (Vorster et al. 2019; Yamada et al. 2020).

In rice, there are five *VPE* (*OsVPE*) genes in the genome (Christoff et al. 2014). Among them, *OsVPE1*, *OsVPE2*, *OsVPE3* and *OsVPE4* were cloned and phylogenetically analyzed (Deng et al. 2011). *OsVPE1* and *OsVPE3* are more like the embryo-type, while *OsVPE2* and *OsVPE4* are more like the vegative organ type (Deng et al. 2011; Lu et al. 2016). OsVPE1 acts as a cysteine protease that cleaves 57-kDa glutelin precursor in the storage vacuolar of rice seeds into acidic and basic subunits (Wang et al. 2009). The expression of *OsVPE2* and *OsVPE3* but not *OsVPE1* or *OsVPE4* was up-regulated by hydrogen peroxide stress (H_2_O_2_) and salt stress (NaCl) (Deng et al. 2011; Kim et al. 2014). Furthermore, the stress-induced enhancement of *OsVPE2* and *OsVPE3* expression, as well as the cell death associated with vacuolar rupture was alleviated by the overexpression of *Bcl-2*, a human apoptotic suppressor (Deng et al. 2011; Kim et al. 2014). *OsVPE3* promotes vacuole rupture and fragmentation of genomic DNA during salt stress-induced PCD, while reducing the leaf width and stomatal guard cell length (Lu et al. 2016). Moreover, the VPE inhibitor inhibited the fusion of vacuoles and delayed the progression of PCD in the aleurone layers (Zheng et al. 2017). *OsVPE3* mediates the initiation of gibberellin (GA)-induced PCD in rice aleurone layers through interacting with actin microfilaments and endogenous H_2_O_2_ (Zhang et al. 2020; Xiao et al. 2021). However, whether or how the rice VPE family members are involved in chilling response and tolerance regulation remains largely unknown.

In this work, we identified *OsVPE2* as a novel chilling responsive gene, whose functional disruption enhances rice chilling tolerance by reducing electrolyte leakage, ROS and MDA accumulation, and enhancing antioxidant enzyme activity without loss of yield. *OsVPE2* is involved in the transcriptional regulatory network of ROS metabolism and DREBs-dependent pathway. Our results indicate that loss-of-function in *OsVPE2* helps protect the vacuole from collapsing under cold stress. The natural variation of *OsVPE2* may confer cold tolerance discrepancy between *japonica* and *indica* rice. These findings suggested that *OsVPE2* is a potential candidate for enhancing chilling tolerance in rice.

## Materials and Methods

### Plant Materials and Phenotypic Analysis

The rice variety Xiangzaoxian31 (XZX31) was used in this study. XZX31 is an early-*indica* conventional rice variety with high-quality cultivated by Rice Research Institute of Hunan Academy of Agricultural Sciences. It is mainly planted in Hunan and Jiangxi provinces, which are the main double-season rice growing provinces in southern China (https://www.ricedata.cn/variety/). Chilling treatment was performed as described previously (Lu et al. 2021). To evaluate chilling tolerance at the seedling stage, surface sterilized seeds were socked in distilled water for 3 d at 30℃. Germinated seeds were sown in the rice seedlings boxes in a light incubator, with a temperature of 28℃, a light intensity of 30,000 lx, a light cycle of 12/12 h (light/dark), and a relative humidity of 70%. Seedlings were cultured in Kimura B solution until the three-leaf stage for low-temperature (10℃) treatment (Ma et al. 2001). The duration of treatment was determined by the time point at which different genetic materials exhibited differences in the degree of leaf curling. For survival rate analysis, the overexpression lines and WT were chilling-treated for 5 d, whereas *OsVPE2*-knockout mutants and WT were chilling-treated for 7 d, and then were recovered at normal temperature for 7 d. Each experiment was biologically repeated at least three times. For the investigation of yield traits, at least 10 plants were examined for each line. The plant materials were potted on the campus of Hunan Agricultural University in 2022.

### Plasmid Generation and Genetic Transformation

To construct the overexpressing vector, a 1506 bp coding sequence (CDS) of *OsVPE2* was amplified from XZX31 and inserted into the binary vector pCAMBIA1300, which was previously modified to contain an ubiquitin promoter. The vector was introduced into *Agrobaterium tumefaciens* strain EHA105 and transformed into XZX31 by *Agrobacterium*-mediated transformation. Four homozygous transgenic lines were obtained, two (OE-1 and OE-2) of which with the highest expression levels of *OsVPE2* were selected for further analysis. The knockout mutants for *OsVPE2*, *osvpe2-1* and *osvpe2-2*, were generated with CRISPR/Cas9 in XZX31 background (BioRun Co. Ltd. Wuhan, China). The primers for amplifying *OsVPE2* CDS and identifying knockout mutants were listed in Table S1.

### Measurement of Physiological Index

For the physiological index measurement in this study, rice seedlings were exposed to chilling stress (10℃) for 3 d and then harvested, and those in a light incubator at 28℃ were served as controls. The DAB (3,3-diaminobenzidine) and NBT (nitrotetrazolium chloride) staining was conducted referring to the method of Kaur et al. (2016). The content of hydrogen peroxide (H_2_O_2_) and superoxide anion (O_2_^−^) of low temperature treated and control plants were measured using kits (Cat No. BC3590 and BC1290, Solarbio, Beijing, China). The electrolyte leakage of chilling-treated and the control plants were measured by the conductivity meter (DS-11 A). The thiobarbituric acid (TBA) colorimetric method was used to determine the content of MDA. The activity of superoxide dismutase (SOD) was determined by riboflavin nitro blue tetrazolium method, and the activity of peroxidase (POD) was determined by guaiacol method. The methods for determining relative conductivity, MDA content, and antioxidant enzyme activity are based on the previous method with slight modifications (Zhou et al. 2022).

### Quantitative Real-time PCR Analysis

The WT seedlings were treated with low-temperature (10 ℃) for 0 h, 12 h, 24 h, 36 h, 48 h, 60 and 72 h to investigate the expression pattern of *OsVPE2* in response to chilling treatment. The overexpression lines and knockout mutants of *OsVPE2* together with WT were treated for 0 and 36 h to analyze the expression of marker genes affected by *OsVPE2*. Total RNA of every sample was extracted using the RNA easy isolation reagent (R701-01, Vazyme, Nanjing, China) and was reverse transcribed for quantitative PCR analysis using the HiScript II qRT Super Mix Kit (R223-01, Vazyme, Nanjing, China). Rice *OsActin* was used as an internal reference. The primers for quantitative Real-time PCR were listed in Table S1. The relative changes in gene expression levels were quantitated based on three biological replicates via the 2^−∆∆Ct^ method.

### Transmission Electron Microscopy (TEM)

The third leaves of the chilling-treated (10℃ for 4 d) seedlings and the control were fixed in 2% (v/v) glutaraldehyde and 3% paraformaldehyde with a phosphate-buffered solution (PBS, pH 7.2) overnight at 4℃ and were then washed with PBS three times. The samples were post-fixed in 1% osmium for 2 h before washing three times with PBS at room temperature. The samples were then passed through a series of alcohol solutions, dehydrated, and embedded in Epon 812 (SPI Supplies, West Chester, PA, USA). The samples were cut into thin sections and stained as described previously with minor modification (Deng et al. 2011). After staining, all sections were examined with a transmission electron microscope (HT7800, Hitachi TEM system, Tokyo, Japan) at 80 kV.

### Haplotype Analyses

Based on publicly available resequencing data from the rice 3K project, nucleotide variations in the *OsVPE2* genomic sequence, including the 2-kb promoter, all exon and intron sequences, and 1-kb 3’ untranslated region, were investigated (RFGB, http://www.rmbreeding.cn/Index/s) (Wang et al. 2018). Sequences of 4084 accessions were used to search for variations in gene using RiceVarMap v2.0 (http://ricevarmap.ncpgr.cn/). For haplotype network analysis, only variation with primary allele frequency ≤ 80% and the deletion frequency ≤ 8% were used for haplotype network analysis. Low-temperature seedling survivability (LTSS) analysis for rice varieties with different haplotypes was conducted as described in the phenotypic analyses. Seedlings were treated at 10℃ for 7 days and survival was counted after recovery. Geographical distribution map of the first six haplotypes was plotted by an online platform (https://www.bioinformatics.com.cn) for data analysis and visualization. Considering the clarity of the diagram, according to the proportion of various haplotypes in different regions, only 1296 and 192 materials were selected for the world and China geographical distribution map, respectively.

## Results

### *OsVPE2* Responds to Chilling Stress and Negatively Regulates Chilling Tolerance

**Fig. 1:**
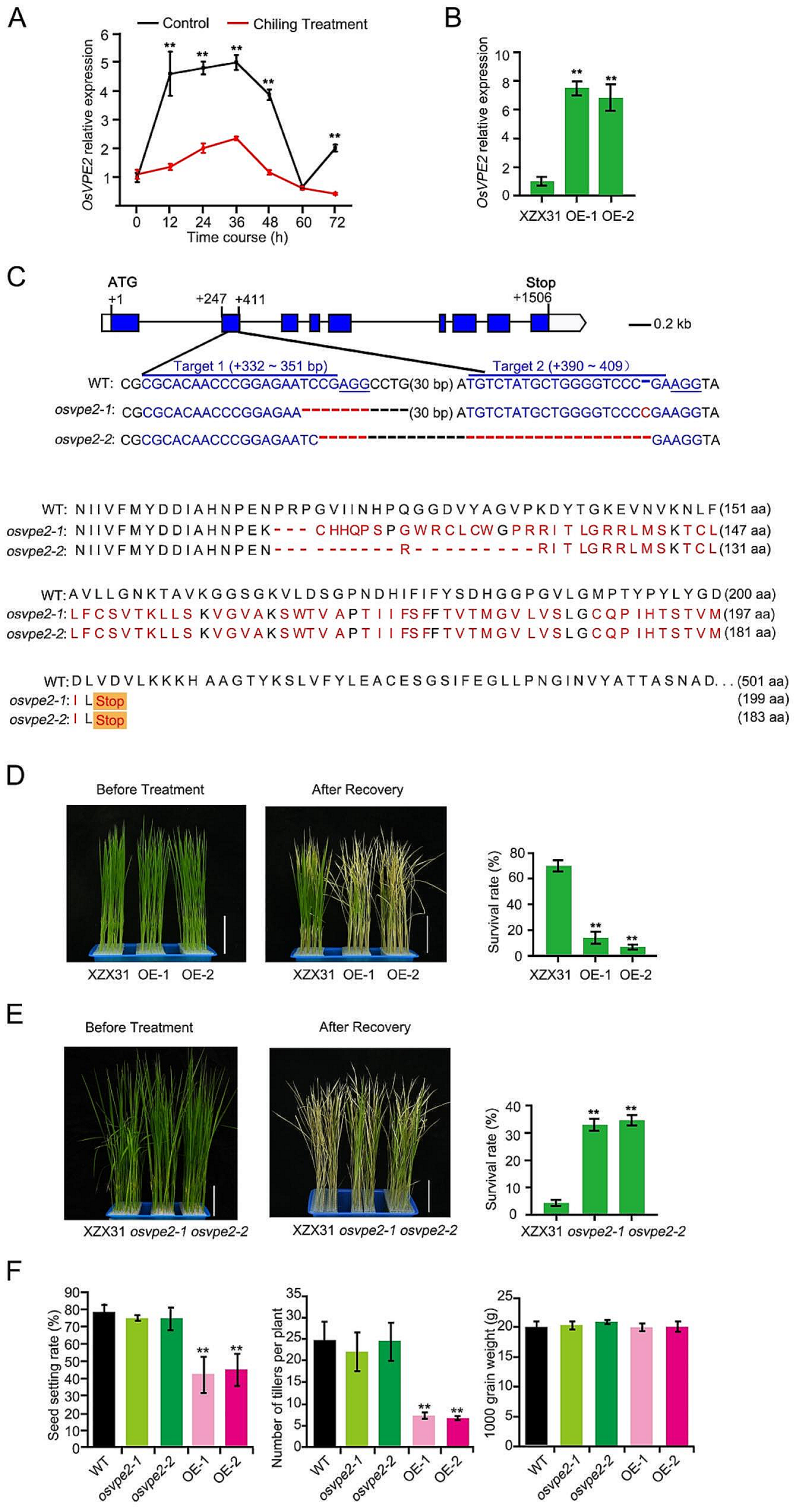
Response of *OsVPE2* expression to low temperature and its regulation of chilling tolerance and yield characters. **A***OsVPE2* gene expression pattern in response to chilling treatment. The value of “0 h” was set to 1. **B** Relative expression levels of *OsVPE2* overexpression lines (OE-1 and OE-2) and wild-type XZX31. The expression level in the wild-type XZX31 was set to 1. **C** CRISPR/Cas9 mediated mutagenesis of *OsVPE2*. Top panel: gene structure of *OsVPE2*. Exons, introns and the 5’- and 3’-UTRs are represented by blue boxes, black lines and open boxes, respectively. The bottom panel shows alignment of WT and *OsVPE2*-knockout mutants’ sequences. The sgRNA targets are highlighted in blue and protospacer-adjacement motif (PAM) sequences are indicated in blue font and underlined. The missing bases of sgRNA targets and PAM sequences in the knockout mutants are represented by red dash-lines while other missing bases are indicted by black dash-lines. The inserted bases in mutants are highlighted in red font, while the corresponding position in WT of the inserted base is represented by a blue short line. The sequence gap length is shown in parentheses. **D** The evaluation of protein translation in *OsVPE2*-knockout mutants along with WT. The font in red color shows variation in amino acids (aa), whereas short-dash lines indicate missing amino acids. The ellipsis at the C-terminus of the WT OsVPE2 protein represents more amino acids up to 501 aa, while the Stop mark at the end of the mutants’ protein sequences represents early termination. **E-F** The chilling tolerance phenotype and survival rates of *OsVPE2*-overexpressing and knockout mutant seedlings, respectively. Bar, 5 cm. Mean and stand deviation are given by three independent experiments. **G** Seed setting rates, number of tillers per plant and grain weight of the above plant materials. ^* *^P < 0.01, (compared with WT; Student’s t-test)

To explore the role of *OsVPE2* in chilling stress responses, its expression pattern in the shoots of the early *indica* rice variety XZX31 at the three-leaf stage in response to chilling treatment was assessed in detail. Compared with seedlings continuously grown at normal temperature (28 °C) for the same time, *OsVPE2* expression levels were significantly decreased in seedlings treated with 12 h, 24 h, 48 h, and 72 h of cold stress (10℃), ranging from 52.7 to 78.3% (Fig. 1A). Although there was no significant difference in *OsVPE2* expression levels between the 60 h cold-treated seedlings and the control, these results demonstrated that *OsVPE2* expression is often inhibited by chilling stress.

To investigate the function of *OsVPE2* for chilling tolerance, two independent homozygous overexpression lines OE-1 and OE-2 were obtained, with *OsVPE2* expression levels were about 6.5 times and 5.8 times higher than that of wild-type (WT), respectively (Fig. 1B). The seedlings of the overexpression lines were subjected to chilling treatment (10 ℃, 5 d) and showed increased chilling sensitivity (Fig. 1E). The survival rate of OE-1 and OE-2 was 14.2% and 5.5%, respectively, which was significantly lower than that of WT (69.7%) (Fig. 1E). Furthermore, two independent knockout lines, *osvpe2-1* and *osvpe2-2* in XZX31 background, were generated through CRISPR/Cas9 mediated gene editing. The mutation in *osvpe2-1* caused 11 bp-deletions from + 348 bp to + 358 bp and 1 bp-insertion at + 407 bp, while *osvpe2-2* mutant contained 58 bp-deletions from + 350 bp to + 407 bp of the coding sequence (Fig. 1C). Both mutations resulted in frame-shift, premature stop codons and thus truncated proteins (501 amino acids in WT; 199 amino acids in *osvpe2-1*; 183 amino acids in *osvpe2-2*) (Fig. 1D). After 7 d of chilling treatment, the survival rates of *osvpe2-1* and *osvpe2-2* were about 32.9% and 33.1%, respectively, while the survival rate of WT was only about 4.2% (Fig. 1G).

To evaluate the application value of utilizing *OsVPE2* to create cold tolerant early-*indica* rice varieties without yield loss, we further investigated the yield traits of *OsVPE2* gene-editing lines and overexpression lines. It was shown that the *OsVPE2* deficiency in the knockout mutants did not affect the seed setting rate, the number of tillers per plant or grain weight, although the overexpression of *OsVPE2* decreased the seed setting rates and number of tillers per plant significantly (Fig. 1F). It was indicated that the *OsVPE2* disruption not only improves the cold tolerance of early-*indica* rice seedlings, but also have no adverse impact on the yield.

### *OsVPE2* Induces ROS Overproduction upon Chilling Stress

**Fig. 2:**
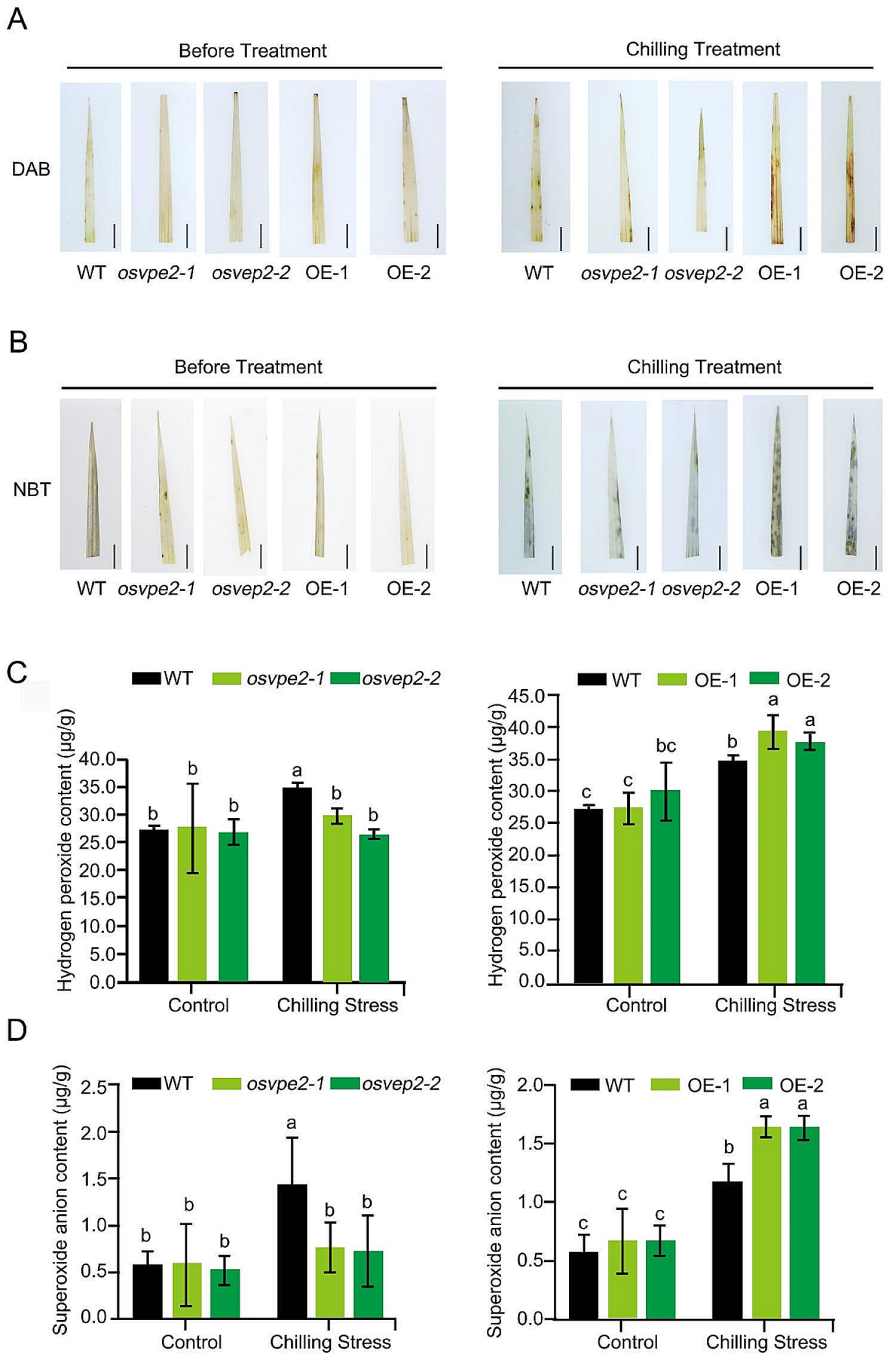
Accumulation of H_2_O_2_ and O_2_^-^ in seedings under chilling stress. **A** DAB staining and **B** NBT staining of leaves before treatment or upon chilling exposure. **C** Quantitative measurement of total H_2_O_2_. **D** Quantitative measurement of total O_2_^-^ content. Bar, 0.75 cm. Data are means ± SD (*n* = 3), Different letters indicate significant differences among all the materials (WT, *OsVPE2*-knockout mutants: *osvpe2-1* and *osvpe2-2*; the overexpression lines of *OsVPE2*: OE-1 and OE-2) under the control and chilling stress treatments by two-way analysis of variance (*p* < 0.05)

When plants encounter low temperature stress, excessive production of ROS such as the H_2_O_2_ and O_2_^-^, which in turn damage cell homeostasis, shatter the cellular membrane and lead to cell death (Mittler et al. 2011). To explore whether *OsVPE2* regulates chilling tolerance through altering ROS homeostasis, the production of H_2_O_2_ and O_2_^-^ was investigated. Before treatment, the ROS staining of WT, knock-out mutants and overexpression lines with DAB (for H_2_O_2_) and NBT (O_2_^-^) solution showed that there were no significant difference among all the materials. However, after chilling treatment, the intensity of staining was lower in knockout mutants but higher in the overexpression lines, compared with WT (Fig. 2A and B). Consistently, the quantitative values of H_2_O_2_ and O_2_^-^ in all the plant materials exhibited no significant difference before treatment (Fig. 2C and D). The H_2_O_2_ accumulation of the overexpression lines exposed to chilling stress increased significantly more than that of WT, however, knock-out mutants showed no-significant increase (Fig. 2C). Under chilling treatment the O_2_^-^ content in both OE-1 and OE-2 was about 1.40 times higher than that in WT, while that in *osvpe2-1* and *osvpe2-2* was only 52.9% and 50.9% of that in WT, respectively (Fig. 2D). These results showed that in response to chilling stress, the harmful H_2_O_2_ and O_2_^-^ in the *OsVPE2-*knockout mutant can be maintained at a lower level, and the accumulation of these harmful substances was higher due to overexpression of *OsVPE2*. It was indicated that the disruption of *OsVPE2* alleviates the production of ROS induced by chilling stress.

### *OsVPE2* Exacerbates Cell Electrolyte Leakage and MDA Accumulation upon Chilling Stress

**Fig. 3:**
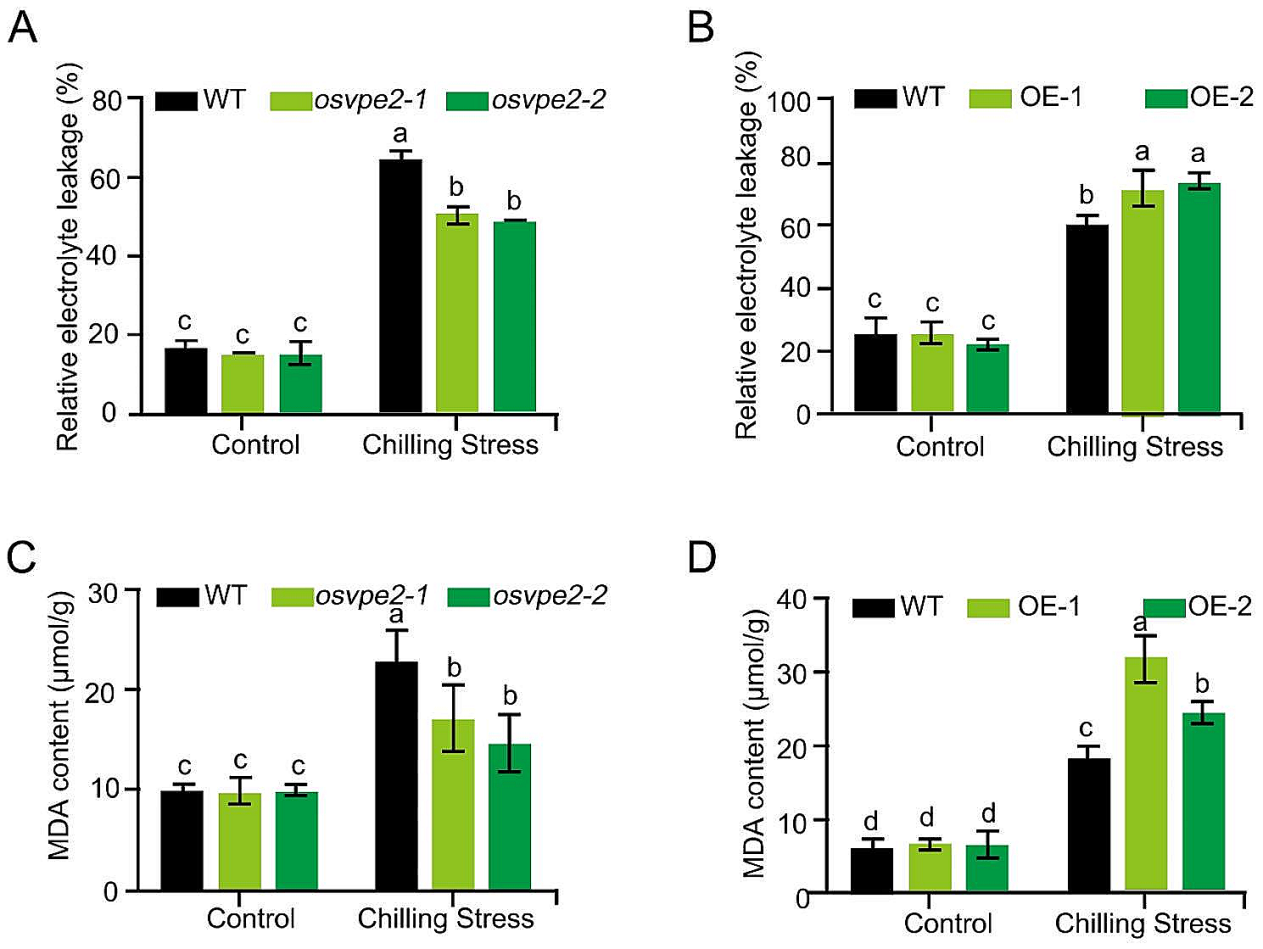
Analysis of the effect of *OsVPE2* on EL and MDA content upon chilling stress. **A** Quantitative measurement of relative EL in WT and *OsVPE2-*knockout mutants. **B** Quantitative measurement of relative EL in WT and the overexpression lines of *OsVPE2*. **C** Quantitative measurement of MDA content in WT and *OsVPE2-*knockout mutants. **D** Quantitative measurement of MDA content in WT and the overexpression lines of *OsVPE2*. Control: normal temperature; Chilling stress: low temperature treatment. Data are means ± SD (*n* = 3). Different letters indicate significant differences among plant materials by two-way analysis of variance (temperature and plant materials) (*p* < 0.05)

Electrolyte leakage (EL) and MDA are the markers of cell membrane injury caused by abiotic stress. To detect whether *OsVPE2* affects the cell membrane integrity under chilling stress, the relative electrolyte leakage and MDA content were measured. As expected, chilling stress resulted in an increase in the relative EL and MDA content of all tested rice materials. When growing at normal temperature, neither the deficiency nor overexpression of *OsVPE2* affects EL and MDA accumulation (Fig. 3). However, under low temperature stress, the relative EL in *OsVPE2* deficient mutants were 21.9–24.3% lower than those in WT (Fig. 3A). Conversely, the relative EL in the overexpression lines are 20–25% higher than those in WT (Fig. 3B). Likewise, the *osvpe2-1* and *osvpe2-2* mutants accumulated 25.1% and 35.7% less MDA than WT, respectively (Fig. 3C). The MDA content in OE-1 and OE-2 were 1.7 and 1.3 fold of that in WT, respectively (Fig. 3D). It was suggested that *OsVPE2* promotes the process of cell membrane damage and MDA accumulation under chilling stress.

### *OsVPE2* Inhibits Antioxidant Enzyme Activities under Chilling Stress

**Fig. 4:**
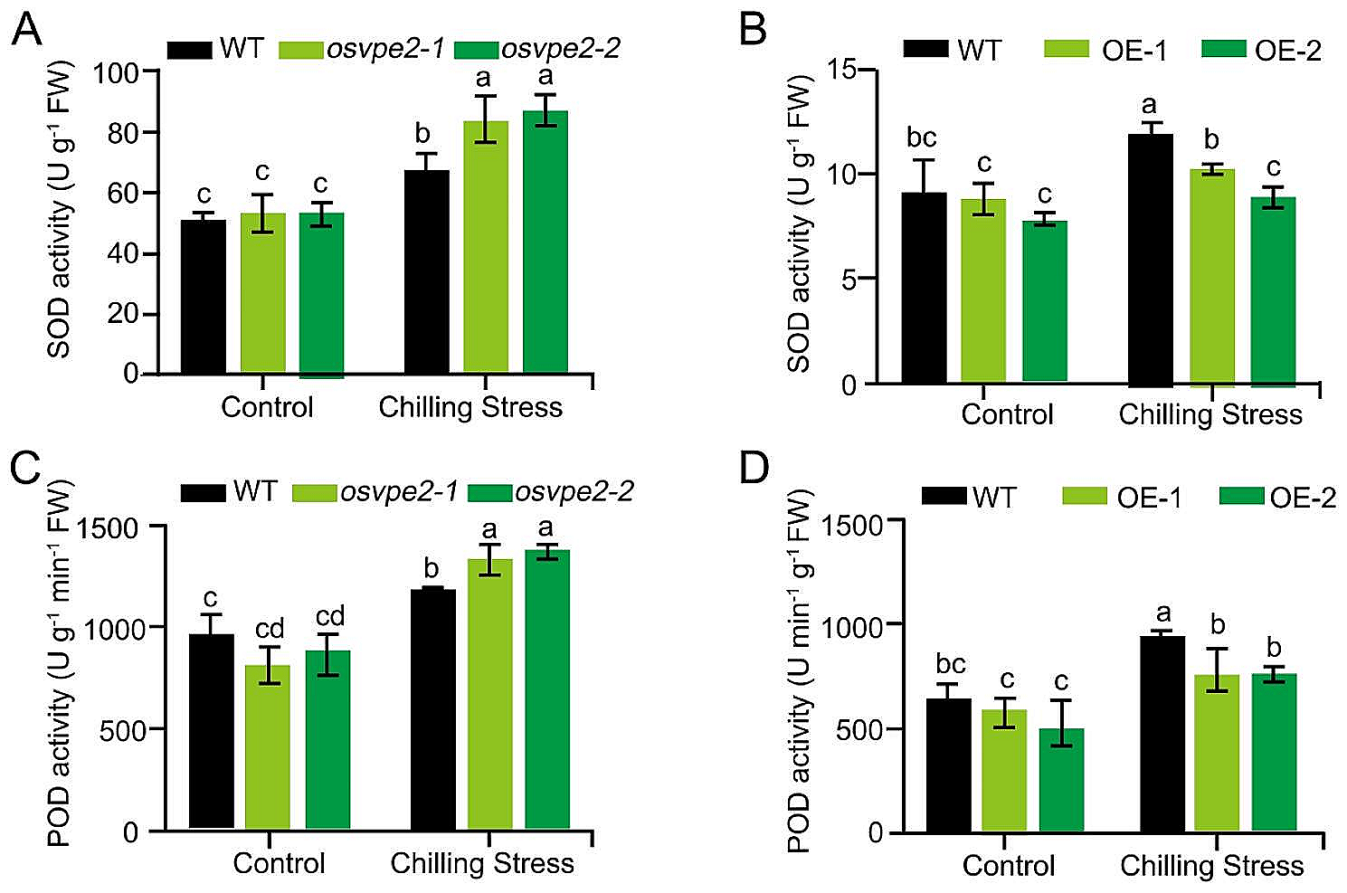
Analysis of the effect of *OsVPE2* on SOD and POD activities upon chilling stress. **A** Quantitative measurement of SOD activity in WT and *OsVPE2-*knockout mutants. **B** SOD activity in WT and *OsVPE2* overexpressing lines. **C** Quantitative measurement of POD activity in WT and *OsVPE2-*knockout mutants. **D** POD activity in WT and *OsVPE2* overexpressing lines. Control: normal temperature; Chilling stress: low temperature treatment. Data are means ± SD (*n* = 3). Different letters indicate significant differences among plant materials by two-way analysis of variance (temperature and plant materials) (*p* < 0.05)

Rice is employed with a sophisticated antioxidant defense system involving a series of antioxidant enzymes to detoxify overabundant ROS produced under cold stress (Li et al. 2022). To investigate the role of *OsVPE2* in ROS scavenging, the enzyme activities of two main antioxidant enzymes including SOD and POD were detected. There was no significant difference between the SOD activities in WT and *OsVPE2*-knockout mutants before chilling treatment. Low temperature caused the increase of SOD activity, but the increase was significantly greater in the knockout mutants, with an increase of 57.9% in *osvpe2-1*, 65.8% in *osvpe2-2*, but only 30.4% in WT (Fig. 4A). However, the SOD activity in the overexpression lines of *OsVPE2* under chilling stress treatment was significantly lower than that in WT (Fig. 4B). Similarly, although there was no significant difference in POD activity among different materials at normal temperature, the *osvpe2* mutants showed significantly higher POD activity than WT upon chilling stress, while the overexpression of *OsVPE2* resulted in significantly lower POD activity (Fig. 4C, D). These results suggested that *OsVPE2* probably inhibits the increase in antioxidant enzyme activity in response to chilling stress, weakening the ROS scavenging and self-protection process in rice.

### *OsVPE2* Affects the Expression of Antioxidant and Stress Tolerance-related Genes

**Fig. 5:**
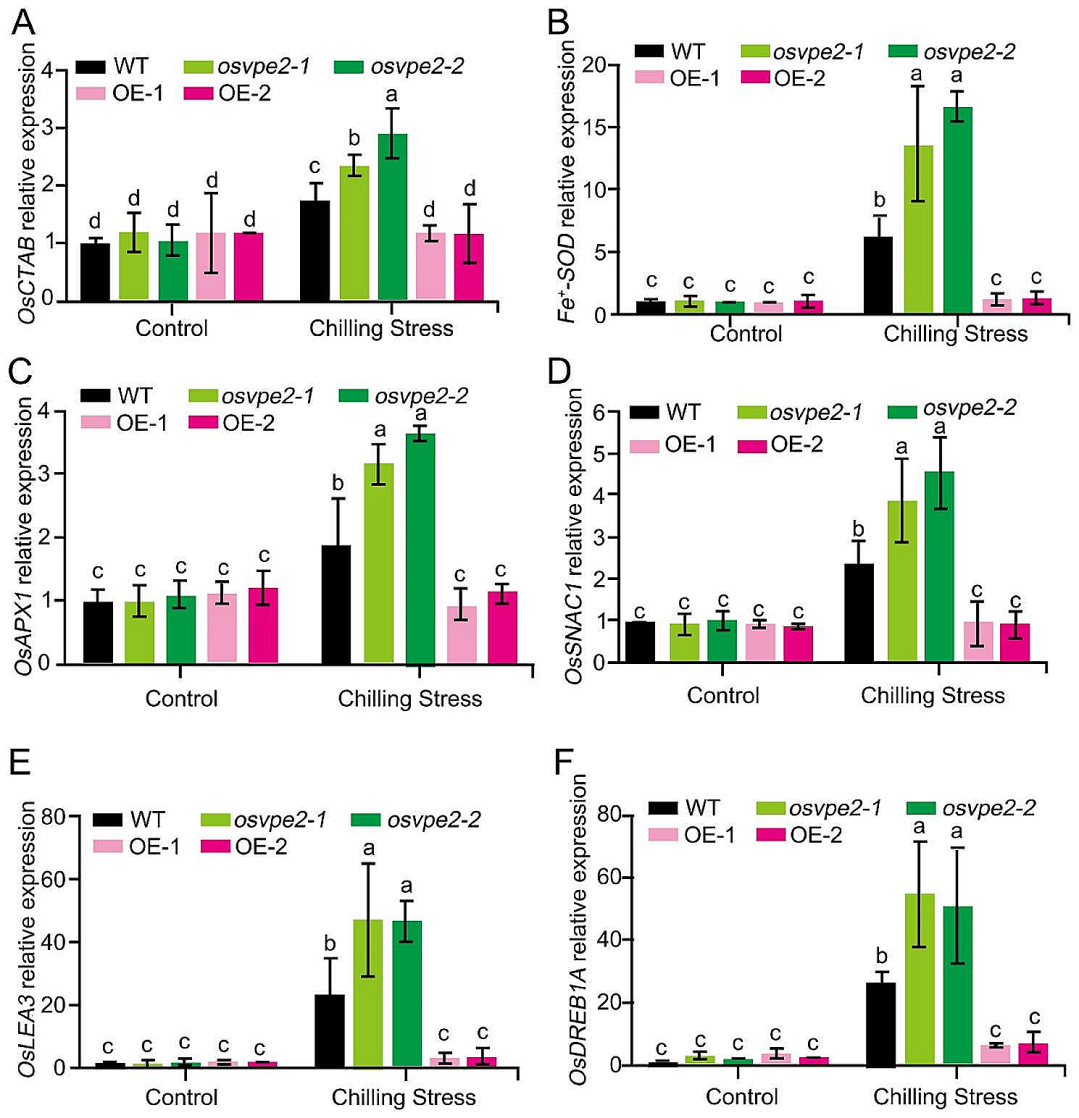
The regulation of *OsVPE2* on expression profile of genes for antioxidant and chilling stress tolerance. **A***OsCTAB* relative expression. **B***Fe+-SOD* relative expression. **C***OsAPX1* relative expression. **D***SNAC1* relative expression. **E***OsLEA3* relative expression. **F***OsDREB1A* relative expression. Data are means ± SD (*n* = 3). Different letters indicate significant differences among plant materials by two-way analysis of variance (temperature and plant materials) (*p* < 0.05)

OsCAT*B, Fe*^*+*^*-SOD, OsAPX1, SNAC1* and *OsLEA3* (*LATE EMBRYOGENESIS ABUNDANT 3*) have been reported previously to play important positive roles in ROS scavenging and confer abiotic tolerance in rice (Kaminaka et al. 1999; Rosa et al. 2010; Ye et al. 2011; You et al. 2014; Yu et al. 2016; Ni et al. 2022). In order to clarify the molecular mechanism of *OsVPE2* in chilling tolerance regulation, the expression patterns of these marker genes closely related to antioxidant and stress tolerance were analyzed in *OsVPE2* transgenic plants and WT plants under control and chilling stress. The results showed that between the *OsVPE2*-knockout mutants and overexpression lines, there was no significant difference in the expression levels of *OsCATB*, *Fe*^*+*^*-SOD, OsAPX1*, *SNAC1* and *OsLEA3*, compared to WT under normal conditions (Fig. 5A-E). Low temperature significantly upregulated the expression levels of these 5 genes in the *OsVPE2-*knockout mutants and WT, and the increase was significantly greater in the *OsVPE2*-knockout mutants than in WT. However, the expression of these 5 ROS scavenging-related genes in overexpressing lines *OE-1* and *OE-2* did not respond to chilling stress treatment (Fig. 5A-E). These results implied that the *OsVPE2*-knockout mutants were more chilling-tolerant likely because the expression levels of these antioxidant-promoting genes were higher in the knockout mutants when subjected to low temperature adversity, suggesting that *OsVPE2* probably represses the transcription of antioxidant genes.

*OsDREB1A*, an important cold-inducible gene in the classical *CBFs/DREBs* regulon, encodes a transcription factor to control the expression of many downstream cold-responsive genes (*CORs*), thereby promoting cold tolerance in rice (Dubouzet et al. 2003; Ito et al. 2006). *OsVPE2* did not affect the expression of *OsDREB1A* at normal temperature (Fig. 5F). However, chilling treatment significantly increased the expression of *OsDREB1A* in WT and *OsVPE2* knockout mutants, with a greater increase in knockout mutants, but did not alter the expression of *OsDRIB1A* in the overexpression lines (Fig. 5F). It was implied that the inhibition of seedling chilling tolerance by *OsVPE2* is likely due to its suppression of *OsDREB1A* gene expression in response to low temperature, suggesting that *OsVPE2* is probably involved in the classical *CBFs/DREBs* regulon of cold signaling pathway.

### *OsVPE2* Deficiency Alleviates the Chilling-Induced Vacuolar Rupture and Mitochondrial Swelling

**Fig. 6:**
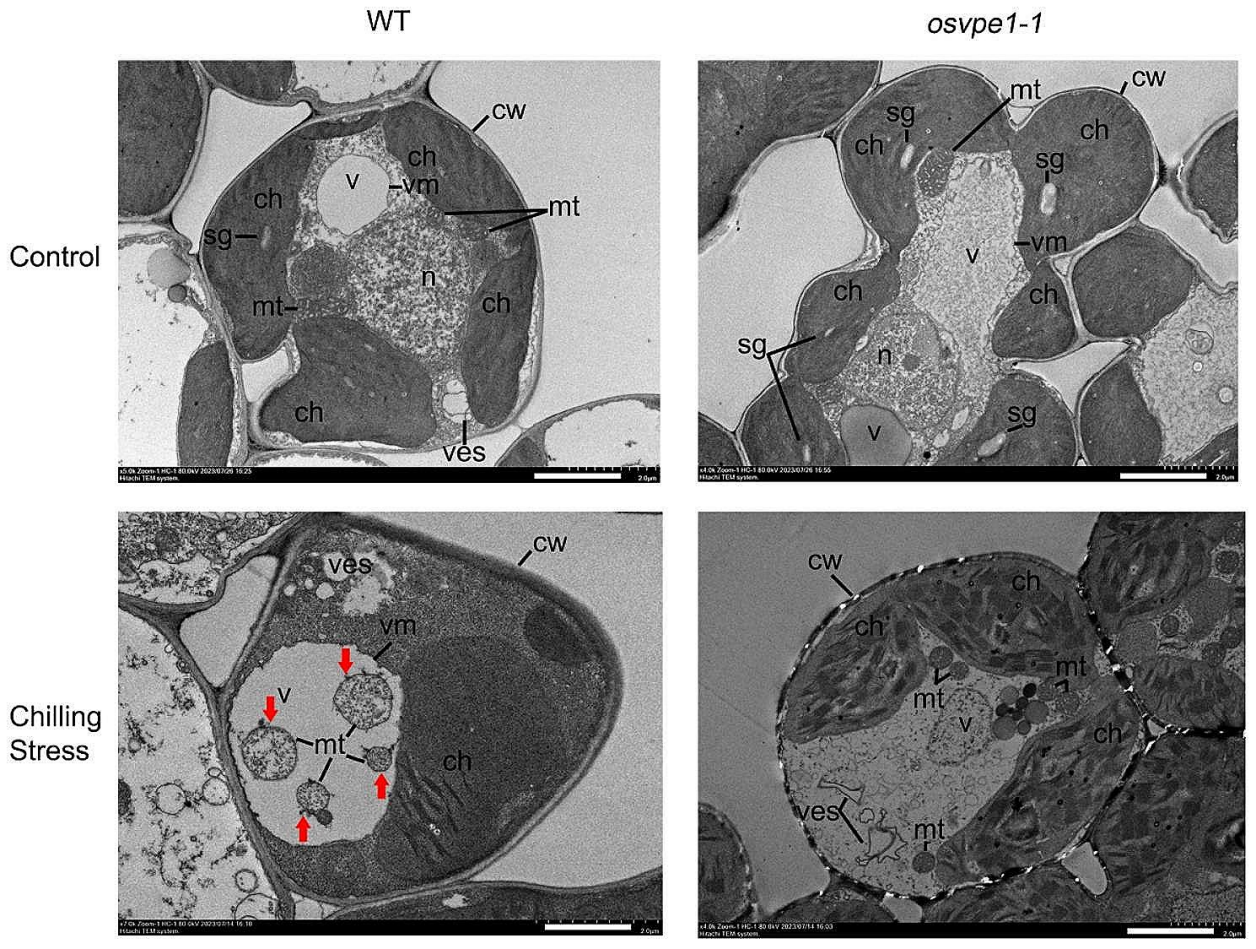
*OsVPE2* deficiency suppresses vacuolar collapse upon chilling stress. cw, cell wall; v, vacuole; vm, vacuolar membrane; ves, vesicles; mt, mitochondrion; n, nuclear; ch, chloroplast; sg, starch granule. Red arrows indicate the abnormal mitochondria. Bar, 2 μm

As the H_2_O_2_ stress induced disruption of the vacuolar membrane (Deng et al. 2011), we investigated the effect of chilling stress on the integration of vacuolar membrane and the role of *OsVPE2* in the disintegration event. As shown by the ultrastructural analysis, chilling stress led to the inclusion of mitochondria in vacuoles (Fig. 6). It was suggested that chilling stress may cause partial collapse of the vacuolar membrane, leading to the mitochondrial entry. Moreover, the morphology of mitochondria was clearly abnormal. Compared with mitochondria in the cytoplasm of control cells from seedlings grown at normal temperature, the mitochondria enclosed in vacuoles upon chilling stress were enlarged (Fig. 6). By contrast, in the *OsVPE2*-deficiency plants *osvpe2-1*, the vacuoles remained intact after chilling treatment as there was no obvious cytoplasmic content such as abnormal organelles into the vacuoles, and no morphological difference of mitochondria were found in the seedlings before and after chilling stress treatment (Fig. 6). These results suggest that *OsVPE2* deficiency is able to prevent vacuolar rupture and mitochondrial swelling in response to chilling stress.

### Natural Variation of *OsVPE2* may Confer Distinct Chilling Tolerance of *Indica* and *Japonica* Rice

**Fig. 7:**
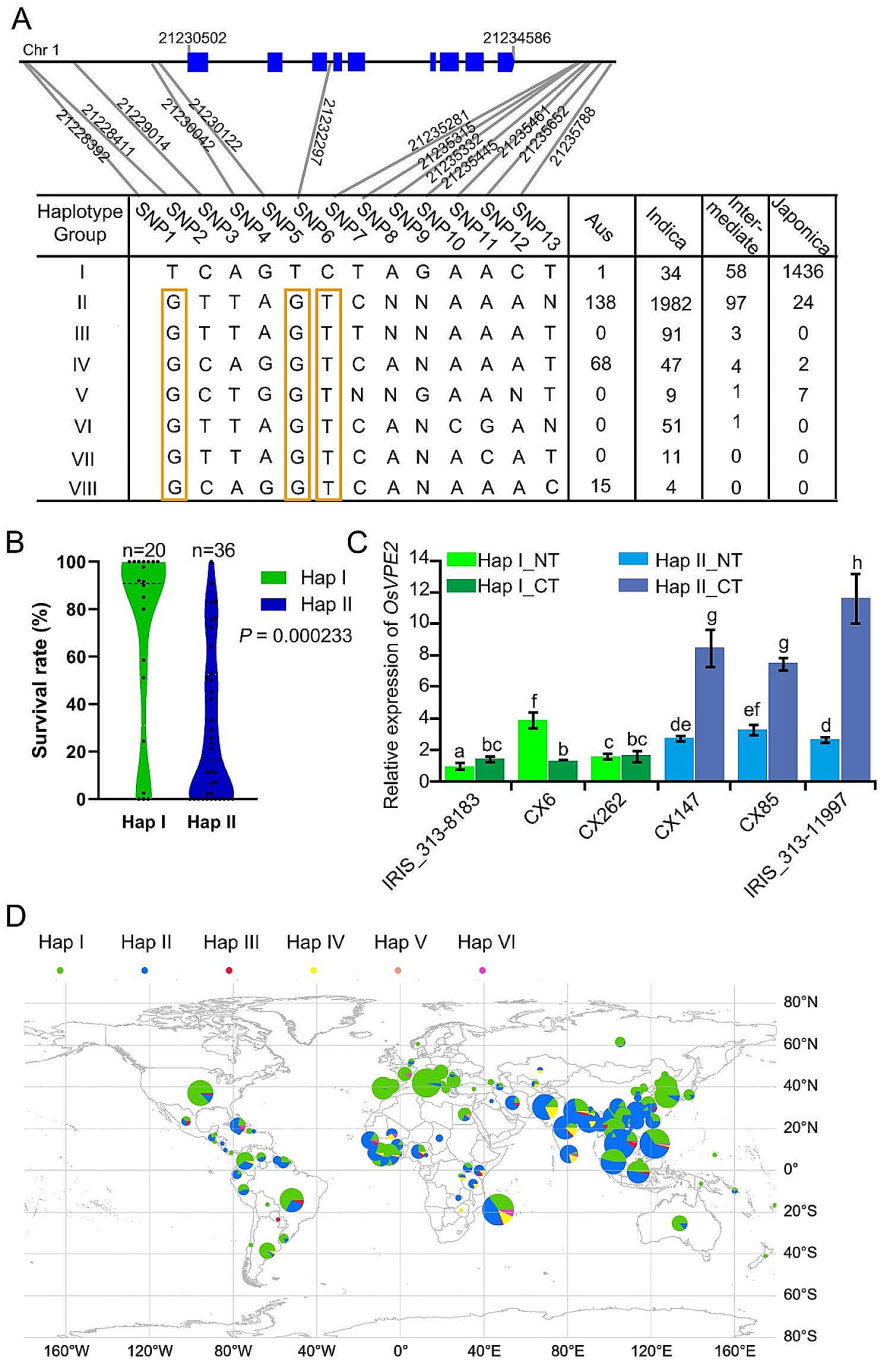
Haplotype analysis of *OsVPE2*. **A** Haplotype analysis of the *OsVPE2* gene region from 4084 rice accessions. The yellow border rectangular box indicates the same differences between Hap II to Hap VIII with Hap (I) **B** The distribution of seedling survival rates upon chilling treatment between Hap I and Hap (II) The bars within violin plots represent medians. **C** Relative expression of *OsVPE2* in representative rice varieties containing Hap I and Hap II. NT, normal temperature; CT, chilling treatment. Data are means ± SD (*n* = 3). Different letters indicate significant differences among plant materials by two-way analysis of variance (temperature and plant materials) (*p* < 0.05). **D** Geographic distributions among 1296 accessions in all over the world. The *x* and *y* axes represent longitude and latitude, respectively

To test for an association between *OsVPE2* natural variation and chilling tolerance in diverse rice germplasm, we performed a haplotype analysis of *OsVPE2* using 4084 rice accessions from the 3 K project (Wang et al. 2018). Five SNPs in the promoter region, one SNP in the 3rd intron and seven SNPs downstream of *OsVPE2* 3’UTR leading to natural variation resulted in eight haplotypes (Hap I to Hap VIII) (Fig. 7A). Hap I is present in 1529 rice accessions, 1436 (93.9%) of which were *japonica* rice varieties. Meanwhile, Hap II is present in 2241 rice accessions, 1952 (87.1%) of which belonged to the *indica* sub-population (Fig. 7A and Fig. S1). The other six haplotypes add up to only 314 accessions, so the main haplotypes are Hap I and Hap II, which are exclusively present in the *japonica* and *indica* sub-population, respectively (Fig. 7A and Fig. S1). *Japonica* rice varieties containing *OsVPE2*^Hap I^, which were selected at random, generally exhibited higher survival rates upon chilling stress than those *indica* rice varieties with *OsVPE2*^Hap II^ (Fig. 7B and Table S2). Notably, Hap II to VIII all have G-G-T sequences at two SNP sites in the promoter region and one SNP site in the intron region, while Hap I has a T-T-C sequence (Fig. 7A). This led us speculate whether the SNP divergence in the promoter region between Hap I and Hap II contributes to the differences in the expression levels of *OsVPE2*. Three chilling-tolerant *japonica* rice varieties with Hap I, and three chilling-sensitive rice varieties with Hap II, were selected to test the expression levels of *OsVPE2*. It was found that *OsVPE2* expression levels in *indica* rice varieties with Hap II were generally higher and increased dramatically upon chilling treatment. The *OsVPE2* expression levels in those *japonica* rice varieties with Hap I were lower and did not show much change or even decreased upon chilling treatment (Fig. 7C). Therefore, Hap I in *japonica* rice is a haplotype with weakened *OsVPE2* function, while Hap II in *indica* rice corresponds to a haplotype with enhanced *OsVPE2* function. Given the role of *OsVPE2* in the negative regulation of chilling tolerance in rice (Fig. 1), we hypothesized that the higher level of expressed *OsVPE2* in *indica* rice with Hap II may be one of the reasons for its lower cold tolerance than *japonica* rice with Hap I.

The geographical distribution analysis further showed that the accessions with Hap I were mainly from high-latitude areas of world such as Korea, Japan and Hungary northern China or high-altitude regions of southwestern China (Yunan-Guizhou plateau with lower average temperatures (Fig. 7C and Fig. S2). The accessions with Hap II were mainly grown in low-latitude areas such as Hunan, Sichuan, and Guangdong Province in South China (Fig. 7C and Fig. S2). It was suggested that the natural variation in *OsVPE2* in different rice subgroups probably contributes to their geographical distribution and environmental adaption.

## Discussion

VPEs were originally named after their roles as the cysteine proteinases responsible for the proteolytic processing and maturation of various vacuolar proteins (Hara-Nishimura and Nishimura 1987; Hara-Nishimura et al. 1991). They are synthesized on the endoplasmic reticulum (ER) as the proprotein precursor and then translocate to vacuoles. In the acidic environment of vacuole the proprotein precursors of VPEs are autocatalytic themselves and may up-regulate other vacuolar hydrolases (Hara-Nishimura and Nishimura 1987; Hara-Nishimura et al. 1991; Rojo et al. 2003). Therefore, VPEs are considered to be the initiators of the vacuolar-processing systems (Hatsugai et al. 2015). In recent years, more and more studies have shown that the plant *VPE* family is involved in the response and tolerance to abiotic stress. For instance, *MdVPEs* of the apple (*Malus pumila*) was induced by abiotic stresses including salinity, cadimium treatment, cold and drought stress (Song et al. 2020). Three out of thirteen genes encoding for *VPEs* in the upland cotton (*Gossypium hirsutum*) showed increased expression upon water logging and salinity (Zhu et al. 2022). The *γVPE* gene in alfalfa (*Medicago sativa*) root was induced by salinity, and the melatonin treatment could decrease *γVPE* gene expression and ROS formation, which in turn prevented salinity-induced PCD (Jalili et al. 2022). In rice, *OsVPE3* mediated salinity-induced PCD and the loss-of-function of *OsVPE3* enhanced the salt tolerance (Lu et al. 2016). However, it remains unknown whether and how the rice *VPE* family is involved in cold response and tolerance regulation.

In this study, our results demonstrated that *OsVPE2* was usually induced by chilling stress (Fig. 1A). The roles of *OsVPE2* have not been reported yet, except for some studies indicating that its expression was increased by hydrogen peroxide and salt treatment (Deng et al. 2011; Kim et al. 2014). Our study is the first to perform phenotypic analysis on *OsVPE2* by constructing overexpression and gene editing lines, thus revealing the important negative role of *OsVPE2* in regulating cold tolerance (Fig. 1). We chose XZX31 as the background material for genetic research, as it can serve as a representative of early rice varieties in typical double cropping rice regions. Improving its cold tolerance at the seedling stage can help increase the planting area of early rice and ensure the total annual rice yield. The *OsVPE2-*knockout mutants generated through the CRISPR/Cas9 gene-editing system exhibited significantly enhanced chilling tolerance. Moreover, the cold tolerance improvement of *OsVPE2* gene-editing lines was not at the cost of yield. There were no significant differences in the main agricultural traits affecting yield when compared to WT (Fig. 1). Moreover, the natural variation in the non-coding region of *OsVPE2* probably confers the distinct levels of *OsVPE2* expression and cold tolerance of *japonica* and *indica* rice (Fig. 7). In the future, we need to further identify the key SNP sites of *OsVPE2* that affect cold tolerance. Knocking out *OsVPE2* by CRISPR/Cas9 technology or utilization of the natural elite alleles of *OsVPE2* to cultivate cold-tolerant varieties will bypass the generation of genetically modified organism, thus alleviating consumer concerns. Our study reveals the great potential of *OsVPE2* as a genetic target for cultivating rice varieties with improved cold tolerance and high yield.

*OsVPE2* contributes to the suppression of antioxidant enzymes SOD and POD activity under cold temperature (Fig. 4). Moreover, upon cold treatment, *OsVPE2* inhibited gene expression of *OsCATB*, *Fe+- SOD*, *OsAPX1*, and *SNAC1*, which are involved in oxidative stress response and ROS clearance (Kaminaka et al. 1999; Rosa et al. 2010; Ye et al. 2011; You et al. 2014; Ni et al. 2022) (Fig. 5). The expression of these genes was induced by low temperature, indicating that rice itself needs to upregulate the expression of these antioxidant genes to eliminate excessive ROS and resist stress. Overexpression of *OsVPE2* inhibited the induction of these genes by low temperature, whereas *OsVPE2* disruption greatly increased the expression level of these genes under low temperature (Fig. 5). It was suggested that *OsVPE2* probably inhibits chilling tolerance through suppression of the antioxidant gene expression. Further investigation is needed on the molecular mechanism by which *OsVPE2* regulates the marker gene expression and antioxidant enzyme activity.

In order to further investigate the physiological mechanism of *OsVPE2* inhibiting cold tolerance in rice seedlings, we conducted sub-cytological examination and found that *OsVPE2* was likely to promote the rupture of vacuole membranes, which was accompanied by mitochondrial entry into vacuoles and mitochondrial swelling (Fig. 6). Previous study on virus-induced hypersensitive cell death mediated by *VPE* in *Nicotiana benthamiana* has showed the disintegration of the tonoplast in the cells of *VPE-*non-silenced plants, whereas in cell of *VPE*-silenced plants, vacuole morphology remained unchanged (Hatsugai et al. 2004). Coincidentally, our study demonstrated that *OsVPE2* promoted the rupture of vacuoles when plants undergo cell death caused by low temperature stress (Fig. 6). The collapse of the vacuole membrane is one of the markers of PCD in plants, and the swelling of mitochondria is consistent with the phenomenon when animal cells undergo PCD (Hatsugai et al. 2015; Shang et al. 2023). Therefore, we speculate that *OsVPE2* may be involved in chilling stress induced PCD, which involves the rupture of vacuolar membranes and changes in mitochondrial morphology. However, more detailed evidence is required in the future to support this hypothesis.

Although our study uncovered the regulatory role of *OsVPE2* in cold tolerance, how *OsVPE2* transduces cold signals remains unknown. One possibility is that cold stress induced *OsVPE2* expression and thus the accumulation of OsVPE2 protein, which localizes in the vacuole and excessive OsVPE2 disrupts the integrity of the vacuole membrane. As reported previously, in rice aleurone layers, OsVPE3 plays a positive role in regulation of the vacuole fusion, which is an important feature of vacuole-mediated PCD in plants (Zhang et al. 2020). OsVPE2 may also affect the vacuole morphology during PCD under low temperature stress, as it has high amino acid similarity with OsVPE3 (68.2%) (Deng et al. 2011). Changes in the morphology of vacuoles and their rupture may further lead to the entry of mitochondria and deformation and functional disorders. The dysfunction of mitochondria in turn results in excessive production of ROS, because it is well-known that ROS is produced by the aerobic metabolism during respiration occurring in mitochondria (Shang et al. 2023). Another possibility is that OsVPE2 transduces the cold signal by causing excessive production of ROS. ROS, as signaling molecules, trigger a series of reactions that ultimately lead to vacuolar membrane rupture, changes in mitochondrial morphology and subcellular localization. As reported before, H_2_O_2_ treatment could result in the disruption of the vacuolar membrane of rice root cells (Deng et al. 2011). These possibilities need to be tested in the future studies.

The mechanism, by which natural variation in the promoter region confers differential transcriptional response and adaptation to environmental factors, has been reported. For example, the 29-bp insertion and/or deletion in the *OsTCP19* promoter can affect the transcriptional repression effect of LBD (LATERAL ORGAN BOUNDARIES DOMAIN) to the *OsTCP19* promoter, thereby conferring divergence in TRN (tillering response to nitrogen) in different rice varieties (Liu et al. 2021). Given that the natural variation in the promoter of *OsVPE2* probably confers the distinct expression levels of *OsVPE2* and cold tolerance of *japonica* and *indica* rice (Fig. 7), it will be interesting to figure out which transcription factor binds directly with *OsVPE2* promoter under chilling stress and how the natural variation in the promoter affects the binding affinity and/or transcriptional activity.

## Conclusions

In this study, the function of *OsVPE2* in negatively regulating cold tolerance was revealed for the first time. *OsVPE2* is likely to promote vacuolar collapse under chilling stress, accompanied by the entry of swollen mitochondria into vacuoles, the overproduction of ROS and MDA, and ultimately cold injury and death of seedlings. Through gene editing technology or the utilization of elite alleles, *OsVPE2* can be made a good target gene for genetic improvement of cold tolerance without yield loss.

### Electronic Supplementary Material

Below is the link to the electronic supplementary material.

Supplementary Material 1. **Table S1**: Primers used in this study. **Table S2**: The low-temperature seedling survivability (LTSS) of rice varieties with the two main haplotypes of *OsVPE2*

Supplementary Material 2. **Figure. S1**: Haplotype network of *OsVPE2* among 4084 rice accessions. Only haplotypes found in more than 10 rice accessions were used to construct the haplotype network. Each circle represents a haplotype and circle size is proportional to the haplotype frequency. Different colors refer to different rice subpopulations.

Supplementary Material 3. **Figure. S2**: Geographic distributions among 192 accessions in China. The x and y axes represent longitude and latitude, respectively

## Data Availability

All data generated or analyzed during this study are included in this published article and its supplemental materials.
