# Supplementary material for: OsVPE2, a Member of Vacuolar Processing Enzyme Family, Decreases Chilling Tolerance of Rice
Source: Rice (N Y). 2024 Jan 9;17:5. doi: 10.1186/s12284-023-00682-9 (PMC10776553; doi:10.1186/s12284-023-00682-9)
Supplement: Supplementary file 1 — Supplementary Material 1. Table S1: Primers used in this study. Table S2: The low-temperature seedling survivability (LTSS) of rice varieties with the two main haplotypes of OsVPE2 [file 12284_2023_682_MOESM1_ESM.docx]

Table S1 Primers used in this study

| **Primer name** | **Primer sequence (5’-3’)** | **Purpose** |
| --- | --- | --- |
| VPE2 HindIII F | CCCaagcttATGGCGGCGCGCGCTCGCCTC | Amplification of CDS |
| VPE2 BamHI R | CGCggatccTTAAGCACTAAAACCCCTATGGGTGG |  |
| VPE2 GE check F | ATGGCCAATCTTGTTTCTGC | Identification of mutants |
| VPE2 GE check R | CCTTGTTGCCTCCAAATTGT |  |
| VPE2 qF | GGCTCCAACGGCTACTACAA | quantitative Real-time PCR |
| VPE2 qR | GCGCGATGTCATCGTACAT |  |
| OsDREB1A qF | CCCTCCATGTGCCTGTAGTT |  |
| OsDREB1A qR | TTCCGCTCCTGACAAACACG |  |
| OsCTAB qF | GTTCGGTTCTCCACAGTCGT |  |
| OsCTAB qR | CCCTCCATGTGCCTGTAGTT |  |
| Fe^+^-SOD qF | CTTGATGCCCTGGAACCTTA |  |
| Fe^+^-SOD qR | GCCAGACCCCAAAAGTGATA |  |
| OsAPX1 qF | CCAAGGGTTCTGACCACCTA |  |
| OsAPX1 qR | CAGTTCGGAGAGCTTGAGGT |  |
| OsSNAC1 qF | CATGGTCCCGTTCTGAGGTG |  |
| OsSNAC1 qR | CACACGTTGCAGCATCGATC |  |
| OsLEA3 qF | TCACTTCAAATTCGGTGCAA |  |
| OsLEA3 qR | CACACCCGTCAGAAATCCTC |  |
| OsActin qF | GACCTTCAACACCCCTGCTA |  |
| OsActin qR | GAGTCCAACACAATACCTGTGG |  |

Table S2. The low-temperature seedling survivability (LTSS) of rice varieties with the two main haplotypes of *OsVPE2*.

| **Genotype Assay_ID** | **Plant name** | **Country** | **Subpopulation** | **LTSS (%)** | ***OsVPE2* Haplotype** |
| --- | --- | --- | --- | --- | --- |
| CX6 | C418 | China | admixed | 100.00 | I |
| CX262 | IAC 3 | Brazil | trj | 100.00 | I |
| IRIS_313-10119 | GITANO::IRGC 82424-1 | Italy | tej | 100.00 | I |
| IRIS_313-8183 | DREW::GERVEX 1650-C1 | United States of America | trj | 97.67 | I |
| IRIS_313-11597 | BHATA PYAGI::IRGC 60895-1 | India | ind3 | 0.00 | I |
| IRIS_313-11129 | HAORU::IRGC 33090-2 | Myanmar | ind3 | 24.39 | I |
| B051 | GZ 1368-5-4 | Egypt | admixed-ind | 0.00 | I |
| IRIS_313-10158 | PICO NEGRO::IRGC 55849-1 | Ecuador | admixed-ind | 0.00 | I |
| IRIS_313-11807 | R 582::IRGC 70504-1 | Colombia | admixed-ind | 2.44 | I |
| CX59 | Milagrosa, Zawa Banday | Philippines | aro | 51.11 | I |
| IRIS_313-10045 | BANTA TIMA::IRGC 69474-1 | Gambia | ind3 | 58.54 | I |
| CX106 | SAL BUI BAO | Viet Nam | trj | 100.00 | I |
| IRIS_313-8285 | KATY::IRGC 77140-1 | United States of America | trj | 100.00 | I |
| IRIS_313-8119 | JUBILIENI::GERVEX 992-C1 | Bulgaria | tej | 100.00 | I |
| B008 | Baxiang | Viet Nam | tej | 91.67 | I |
| IRIS_313-8132 | RUBI::GERVEX 1247-C1 | Portugal | tej | 100.00 | I |
| IRIS_313-11204 | KARTIKHAMA::IRGC 37149-2 | Bangladesh | ind3 | 90.00 | I |
| CX158 | Shwe War Tun | Myanmar | ind2 | 80.00 | I |
| IRIS_313-10235 | PSBRC 68::IRGC 99711-1 | Philippines | ind2 | 85.00 | I |
| IRIS_313-9302 | GAM PAI 30-12-15::IRGC 831-1 | Thailand | admixed-ind | 92.00 | I |
| CX35 | BG 94-1 | Sri Lanka | admixed-ind | 2.22 | II |
| CX85 | C 71 | Viet Nam | admixed-ind | 0.00 | II |
| CX147 | Seberang | Malaysia | ind2 | 0.00 | II |
| CX230 | IR 64-IL | Philippines | ind2 | 100.00 | II |
| CX348 | Zhonghua 1 | China | admixed-ind | 11.54 | II |
| IRIS_313-8341 | BAT DO::IRGC 7014-1 | Viet Nam | admixed-ind | 64.29 | II |
| IRIS_313-9606 | DA 11::IRGC 6046-1 | Bangladesh | ind3 | 0.00 | II |
| IRIS_313-8697 | YEBAWYIN::IRGC 33885-1 | Myanmar | ind3 | 21.28 | II |
| IRIS_313-9551 | BENGALY MORIMO::IRGC 10976-1 | Madagascar | ind3 | 51.06 | II |
| IRIS_313-10109 | EX EBOKOZURU::IRGC 79912-1 | Nigeria | ind2 | 0.00 | II |
| IRIS_313-9590 | KETAN SERANG::IRGC 14615-1 | Indonesia | ind2 | 42.42 | II |
| IRIS_313-10333 | B 6136-3-TB-0-1-5::IRGC 117312-1 | Indonesia | ind2 | 0.00 | II |
| IRIS_313-8453 | ARC 18597::IRGC 43299-1 | India | ind3 | 83.33 | II |
| IRIS_313-10287 | UQUIHUA::IRGC 117037-1 | Peru | ind2 | 76.19 | II |
| IRIS_313-11272 | ARC 14756::IRGC 41736-1 | India | aus | 0.00 | II |
| IRIS_313-11997 | BARUM::IRGC 79108-1 | Cambodia | admixed-ind | 0.00 | II |
| IRIS_313-11437 | CAMPECHE A 79::IRGC 51102-1 | Mexico | ind2 | 33.33 | II |
| IRIS_313-11737 | CHUNDI::IRGC 67486-1 | India | aus | 28.89 | II |
| IRIS_313-11720 | DAW 85::IRGC 66704-1 | Thailand | ind3 | 75.56 | II |
| IRIS_313-10980 | DUDHSAR::IRGC 26458-1 | Bangladesh | admixed-ind | 0.00 | II |
| IRIS_313-11920 | GI TAH::IRGC 74883-1 | Thailand | ind3 | 0.00 | II |
| IRIS_313-12128 | HMA GNENG::IRGC 85751-1 | Lao People's Democratic Republic | admixed-ind | 2.17 | II |
| IRIS_313-11414 | KARUNJEERAGA SAMBA::IRGC 49774-1 | India | ind3 | 0.00 | II |
| IRIS_313-11708 | KHAO YAI GUANG::IRGC 65972-1 | Thailand | admixed-ind | 6.52 | II |
| IRIS_313-10748 | LUA DUC::IRGC 16718-1 | Viet Nam | admixed-ind | 25.71 | II |
| IRIS_313-11762 | NS 1515::IRGC 68947-1 | Madagascar | ind3 | 0.00 | II |
| IRIS_313-11731 | QING TAI AI::IRGC 67273-1 | China | admixed-ind | 50.00 | II |
| IRIS_313-10986 | HALDI JAON::IRGC 26638-2 | Bangladesh | ind3 | 90.91 | II |
| IRIS_313-10614 | HAM MOON::IRGC 9135-2 | Hong Kong | admixed-ind | 45.24 | II |
| IRIS_313-9320 | SIPULUT HITAM PENDEK::IRGC 20154-1 | Indonesia | ind2 | 11.11 | II |
| CX305 | ZH 5 | China | ind2 | 52.94 | II |
| IRIS_313-11787 | TANDAKAY FINGO::IRGC 69793-1 | Gambia | ind3 | 11.36 | II |
| IRIS_313-12270 | TOAM JUM::IRGC 96077-1 | Myanmar | ind3 | 7.14 | II |
| IRIS_313-8751 | HNANWA::IRGC 33118-1 | Myanmar | ind3 | 72.34 | II |
| IRIS_313-11351 | BANCHI::IRGC 45055-2 | India | ind3 | 82.93 | II |
| IRIS_313-12334 | DO KHAW::IRGC 106977-1 | Lao People's Democratic Republic | admixed-ind | 23.53 | II |
