# Supplementary figures and images for: OsVPE2, a Member of Vacuolar Processing Enzyme Family, Decreases Chilling Tolerance of Rice
Source: Rice (N Y). 2024 Jan 9;17:5. doi: 10.1186/s12284-023-00682-9 (PMC10776553; doi:10.1186/s12284-023-00682-9)

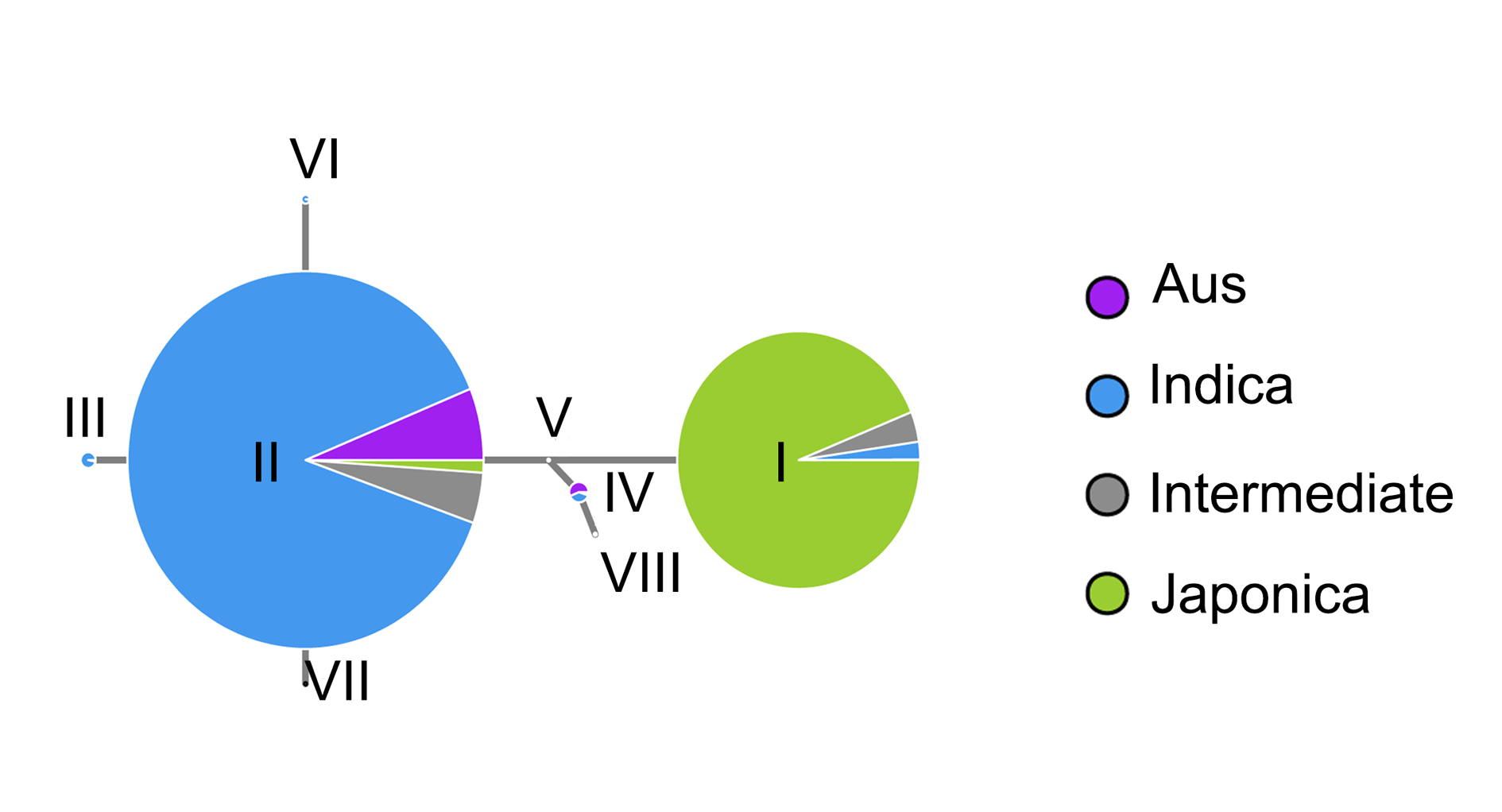

Supplement: Supplementary file 2 — Supplementary Material 2. Figure. S1: Haplotype network of OsVPE2 among 4084 rice accessions. Only haplotypes found in more than 10 rice accessions were used to construct the haplotype network. Each circle represents a haplotype and circle size is proportional to the haplotype frequency. Different colors refer to different rice subpopulations. [file 12284_2023_682_MOESM2_ESM.tif]

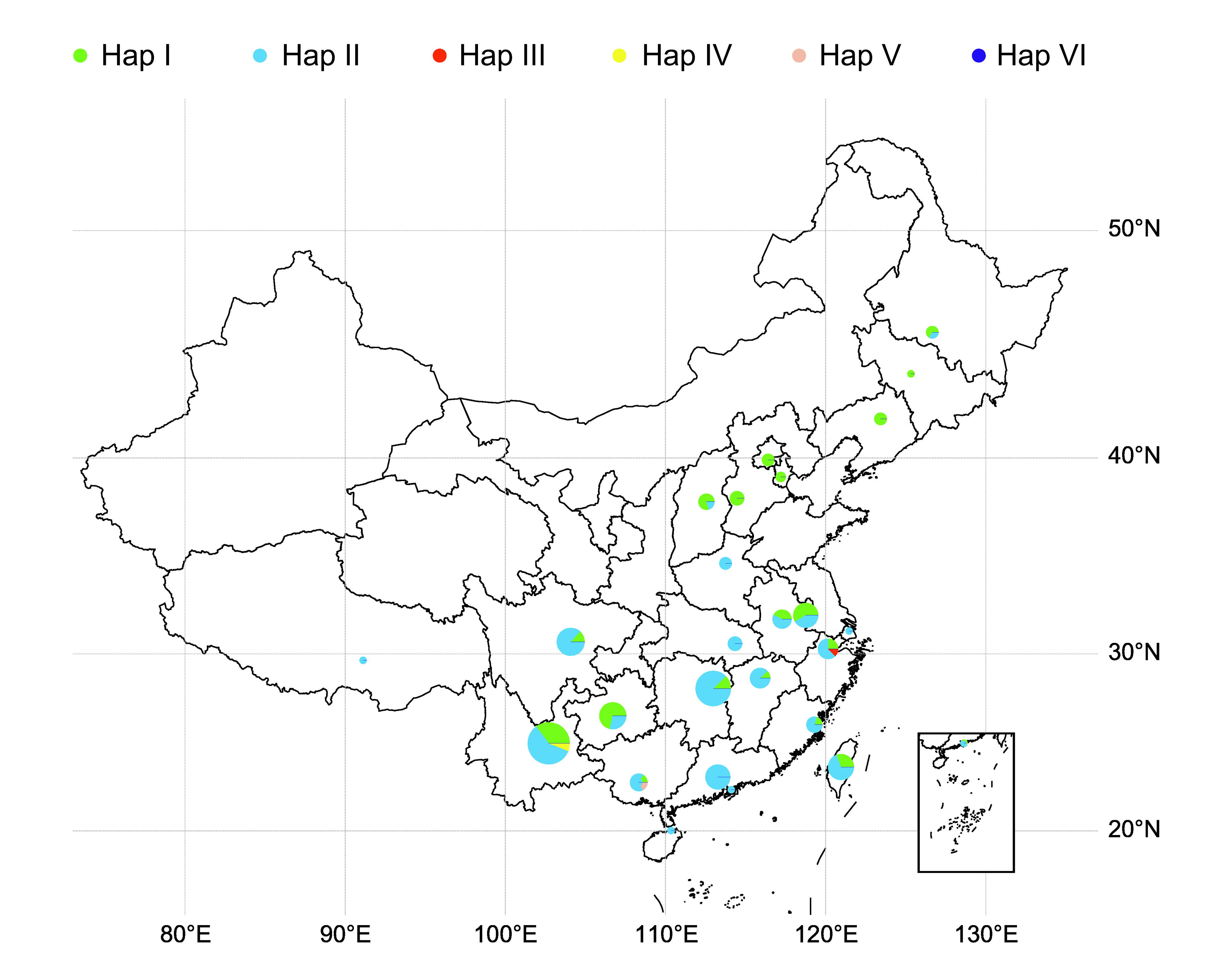

Supplement: Supplementary file 3 — Supplementary Material 3. Figure. S2: Geographic distributions among 192 accessions in China. The x and y axes represent longitude and latitude, respectively [file 12284_2023_682_MOESM3_ESM.tif]
